# Arrhythmic Risk in Syncope: Bridging Guidelines and Real-World Evidence

**DOI:** 10.31083/RCM45722

**Published:** 2025-10-24

**Authors:** Maria Giulia Bellicini, Gianmarco Arabia, Antonio Curnis

**Affiliations:** ^1^Institute of Cardiology, ASST Spedali Civili di Brescia, 25123 Brescia, Italy; ^2^Department of Molecular and Translational Medicine, University of Brescia, 25121 Brescia, Italy

The 2018 European Society of Cardiology (ESC) Guidelines on syncope devote significant attention to excluding sustained ventricular tachycardia (VT) as a possible cause [1]. This editorial provides a guideline-based review focused on the current evidence on arrhythmic syncope in the context of the 2018 ESC Syncope, 2017 ACC/AHA/HRS Syncope, and 2022 ESC VA/SCD guidelines. It is not intended to be a systematic review or meta-analysis.

Observational evidence from large case series and registry studies, indicates that sustained VT—except for Torsades de Pointes (TdP)—is an uncommon cause of self-limited syncope [1, 2, 3, 4]. TdP can self-terminate and is typically associated with congenital or acquired long QT syndromes, most often when QTc ≥500 ms [5]. Other forms of sustained VT, particularly scar-related re-entry, generally lack the instability of TdP and therefore tend not to terminate on their own. Once initiated, these circuits can sustain activity until interrupted, frequently degenerating into ventricular fibrillation and cardiac arrest [6].

Structural heart diseases without mechanical obstruction—such as cardiomyopathy—rarely causes VT-related syncope, which is more characteristic of obstructive lesions such as severe aortic stenosis or hypertrophic cardiomyopathy, which typically trigger exertional syncope and are readily identifiable by examination and echocardiography. Channelopathies, other than long QT syndrome, seldom lead to sustained VT at the time of syncope. Loop recorder data in Brugada and short QT syndromes have confirmed these low event rates [7].

Although guideline-based stratification has improved standardization [1], their broad definitions of abnormal electrocardiogram (ECG) which include conduction delays, axis deviation, left ventricular hypertrophy, and non-specific repolarization changes; often describe findings that are within normal variants, particularly in older adults. Only more pathological findings, such as Q waves or other clear signs of previous myocardial injury, more often indicate structural heart disease. However, in these conditions, sustained VT is not usually associated with self-limited syncope but rather with cardiac arrest.

Non-sustained VT (NSVT) most often reflects transient automaticity or an unstable re-entry that cannot perpetuate, and is seldom responsible for syncope in either structurally normal or diseased hearts [2, 6]. NSVT is not equivalent to sustained VT and usually carries a different clinical prognosis. Post-infarction NSVT often originates from re-entry within a scar that is insufficiently organized to maintain a sustained circuit. As a result, most episodes terminate spontaneously after only a few beats, remain asymptomatic, and do not progress to sustained VT or cardiac arrest [8, 9, 10, 11]. Device diagnostics from Implantable Cardioverter-Defibrillator (ICD) cohorts and implantable loop recorder studies, consistently show that NSVT is more commonly detected as a background finding, and is rarely temporally linked with syncopal episodes, whereas documented events at the time of syncope are more often brady-arrhythmic [1, 12]. This suggests that many NSVTs in the ischemic population reflect a scar substrate insufficiently organized to sustain persistence and therefore carry a lower immediate risk. In cardiomyopathies, NSVT usually arises from diffuse remodelling and fibrosis, producing unstable re-entry or triggered activity that cannot sustain longer periods of tachycardia, which explains its usual short and self-limited nature [2, 6]. In channelopathies like the Brugada syndrome, NSVT is typically absent or benign, with the exclusion of rare exceptions such as catecholaminergic polymorphic VT [5, 13].

Brady-arrhythmias, including paroxysmal atrioventricular (AV) block or long sinus pauses, can cause syncope, but typically reflect progressive conduction system fibrosis. This process evolves slowly, so that the immediate risk of sudden cardiac death is low even when syncope occurs [14]. Many cases are identifiable soon after the event or on short-term monitoring, although implantable loop recorder studies demonstrate that the diagnosis can require prolonged periods of observation [12], most often revealing paroxysmal high-grade AV block and, less frequently, long sinus pauses [7, 12, 15]. While such findings usually trigger pacemaker implantation, the actual benefit to patients experiencing only one or two events per year is debatable.

While prodromes point toward vasovagal or hypotensive etiologies, their absence does not confirm an arrhythmia; since a substantial proportion of non-arrhythmic syncopal episodes also occur without warning signs [2]. However, true syncope is best distinguished from collapse secondary to shock, such as gastrointestinal bleeding or pulmonary embolism which lead to sustained hypotension, in which the loss of consciousness may not spontaneously resolve [1].

In summary, sustained VT—apart from TdP—is an uncommon cause of syncope, in which scar-related sustained VT does not typically self-limit. When the origin is due to an arrhythmia, syncope is more often due to brady-arrhythmias. These events can lead to transient loss of consciousness, but unlike sustained ventricular arrhythmias, they usually do not carry an imminent risk of sudden cardiac death. Most syncopal episodes are benign. Guideline-based evaluation can help avoid unnecessary interventions while focusing resources on the minority who are at genuine arrhythmic risk.

## Abbreviations

AV, atrioventricular; ESC, European Society of Cardiology; ECG, electrocardiogram; ICD, implantable cardioverter-defibrillator; MI, myocardial infarction; NSVT, non-sustained ventricular tachycardia; TdP, Torsades de Pointes; VT, ventricular tachycardia.
